# Sulphurous Mineral Waters: New Applications for Health

**DOI:** 10.1155/2017/8034084

**Published:** 2017-04-06

**Authors:** Jose Manuel Carbajo, Francisco Maraver

**Affiliations:** ^1^Professional School of Medical Hydrology, Faculty of Medicine, Universidad Complutense de Madrid, 28040 Madrid, Spain; ^2^Department of Physical Medicine and Rehabilitation, Medical Hydrology, Faculty of Medicine, Universidad Complutense de Madrid, 28040 Madrid, Spain

## Abstract

Sulphurous mineral waters have been traditionally used in medical hydrology as treatment for skin, respiratory, and musculoskeletal disorders. However, driven by recent intense research efforts, topical treatments are starting to show benefits for pulmonary hypertension, arterial hypertension, atherosclerosis, ischemia-reperfusion injury, heart failure, peptic ulcer, and acute and chronic inflammatory diseases. The beneficial effects of sulphurous mineral waters, sulphurous mud, or peloids made from sulphurous mineral water have been attributed to the presence of sulphur mainly in the form of hydrogen sulphide. This form is largely available in conditions of low pH when oxygen concentrations are also low. In the organism, small amounts of hydrogen sulphide are produced by some cells where they have numerous biological signalling functions. While high levels of hydrogen sulphide are extremely toxic, enzymes in the body are capable of detoxifying it by oxidation to harmless sulphate. Hence, low levels of hydrogen sulphide may be tolerated indefinitely. In this paper, we review the chemistry and actions of hydrogen sulphide in sulphurous mineral waters and its natural role in body physiology. This is followed by an update of available data on the impacts of exogenous hydrogen sulphide on the skin and internal cells and organs including new therapeutic possibilities of sulphurous mineral waters and their peloids.

## 1. Introduction

Hydrogen sulphide (H_2_S), the active molecule in sulphurous mineral waters, is currently attracting the attention of scientists due to its potential therapeutic applications [1]. These past few years have witnessed a growing body of knowledge regarding the potent role of hydrogen sulphide as a signalling molecule and protein sulfhydration [2] in numerous cytoprotective biochemical reactions of the body. Besides supporting the known properties of sulphurous mineral waters, these new data are starting to unveil new therapeutic applications for these waters.

Being a gas, H_2_S can be absorbed by numerous routes. It is able to penetrate the skin and mucosae and can therefore act at the cell level both in the skin and in internal organs of our organism. This means that the topical application of sulphurous mineral waters rich in hydrogen sulphite has the potential to treat disorders of the internal organs such as high blood pressure, ischemia, and conditions affecting the kidneys or nervous system. Further, if sulphurous mineral water is applied to the skin in the form of a matured mud, or peloid, its effects may be potentiated. Many authors have examined the mechanisms of action of medicinal waters and their therapeutic effects, and certain inorganic components have been linked to the effects of curing baths [3–7] and muds [8–11].

Several studies have also assessed the properties of mineral water as a whole, although the different constituents of waters may act in an antagonistic manner [12]. Similarly, peloids have often been considered as equivalent despite their different origins conferring them very different properties [13].

In this paper, we revisit the chemical properties of H_2_S in sulphurous mineral waters and describe how environmental factors such as pH, temperature, and the presence of oxygen can affect its concentrations and thus the final activity of the waters or their products. Finally, we review the impacts of H_2_S on mammalian cells and organs, with special attention paid to the new therapeutic possibilities of sulphurous mineral waters and their peloids.

## 2. Hydrogen Sulphide Chemistry

Hydrogen sulphide is a colourless gas and, being a weak acid, is highly soluble in water. It is heavier than air, very poisonous, flammable, and corrosive. Above a certain concentration, its toxic effects are comparable to those of carbon dioxide and cyanide. Its stability depends on the pH, temperature, and the oxygen concentration of the environment.

Sulphur forms change at two critical pHs, pKa = 7.04 and pKa = 11.96. At physiological pH, the ratio of hydrogen sulphide to bisulphide (HS^−^) is 1 : 3. Thus, two identical solutions of H_2_S show different concentrations at different ambient temperature and the presence of oxygen will promote the reduction of hydrogen sulphide to hydrogen sulphate.

**Figure 1:**
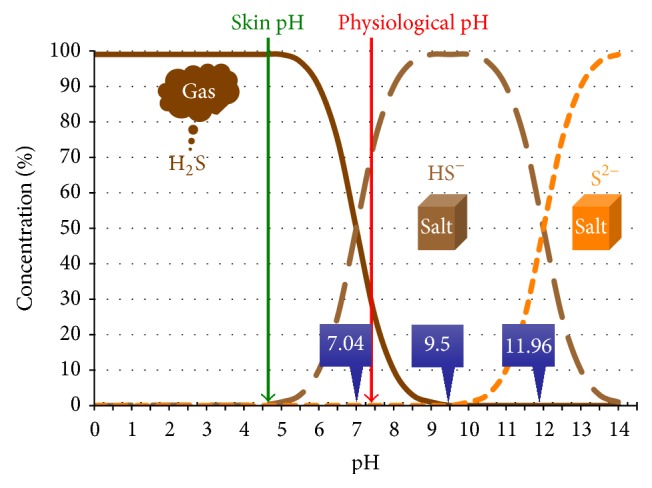
Hydrogen sulphide concentrations versus pH. According to the pH (7.04 or 11.96), sulphurous derivatives occur in saline (HS^−^ and S^2−^) or gaseous (H_2_S) solution. On the skin (pH < 6), hydrogen sulphide is mainly present as gas (H_2_S), whereas, at physiological pH, (7.2–7.4) only one-third exists as gas (H_2_S) and it is mainly found in saline solution (HS^−^).

At acidic pH, H_2_S is the only form of sulphur. At a pH of 7.04, sulphur salts occur at a 50% concentration and when the pH is around 9.5 only bisulphide will exist (HS^−^). Beyond pH 9.5, sulphides (S^2−^) start to form and as pH increases, this anion is the only viable form of sulphur (Figure 1).

At physiological pH (7.2–7.4), only a third of sulphur is found as H_2_S, although, at skin pH (4.5–6.5), virtually all sulphur occurs as H_2_S.

The composition of sulphurous mineral water has been well-established. However, regardless of accompanying salts, for this mineral water to exert its beneficial effects, it must contain a reasonable amount of H_2_S or HS^−^ and thus have a relatively acidic pH. In contrast, S^2−^ usually found in alkaline waters are inert or do not exert the same activity [14].

Another factor to consider is that although H_2_S is a gas, HS^−^ and S^2−^ are salts. This means that H_2_S will be more easily absorbed through the skin and mucosa than its soluble salts. Further, given that the reactivity of dissociated HS^−^ is greater than that of H_2_S, it may be assumed that [15] (1) both the gas and anions coexist in vivo; (2) dissociated bisulphide is more reactive than H_2_S and peaks at pH 11 [16]; and (3) the presence of oxygen could diminish the reduction force. This means that H_2_S molecules will more actively penetrate the skin rather than remaining on the surface.

Hydrogen sulphide derivatives will act depending on their molecular state too, that is, their degree of reduction or oxidation, which is usually determined by the external environment. Oxidation causes the transformation of H_2_S (valence −2) to sulphur (S valence: 0), which is in turn oxidized to hyposulphites (H_2_S_2_O_3_: valence +2) and then to sulphites (H_2_SO_3_: valence +4) and sulphates (H_2_SO_4_: valence +6). Conversely, reduction leads to the reverse order of formation [17].

In nature, H_2_S is generated by the decomposition of organic matter, whereby it is aerobically oxidized to elemental sulphur and then transformed to sulphates through the activity of bacteria and other microorganisms. Likewise, sulphates can be anaerobically reduced to H_2_S through their assimilation by living organisms or their decomposition by microorganisms, thus closing the sulphur cycle [18]. Thus, in simple terms, anaerobically reduced sulphates give rise to H_2_S, which is aerobically oxidized to sulphate.

In balneology, medicinal muds (peloids) can be generated through these reactions [9]. This mud has similar properties to water [19] and matures over time improving their properties of granulometry, specific heat, caloric retentivity, inertia time, relaxation time, hardness, adhesiveness, cohesion, and springiness [20, 21]. Mud treatments have been used for wounds produced by trauma. In this application, water remains in contact with the skin surface for a long time and absorption of the active principle is enhanced due to occlusion and ion exchange at temperatures slightly above physiological [22]. For this purpose, the use of peloids produced from sulphurous mineral waters is common. These peloids have their own peculiar characteristics [11].

According to the data available so far, balneotherapy using thermal-mineral waters and peloids has proved to be an effective remedy for lower back pain and knee and hand osteoarthritis [23–26].

## 3. Hydrogen Sulphide and Body Physiology

Recently, H_2_S and nitric oxide (NO) have been described as gasotransmitters that contribute to many physiological and pathophysiological functions as signalling molecules with potent cytoprotective actions [27].

Hydrogen sulphide is an endogenous gas with important physiological functions [28]. Endogenous hydrogen sulphide has been reported to function as a neuromodulator in the brain [29] and within the vasculature; the main functions of H_2_S are vasodilation and promoting new vessel growth.

The proangiogenic effects of hydrogen sulphide have been associated with increased vascular endothelial growth factor (VEGF) expression and activation of its receptor. Hydrogen sulphide-based therapies have therapeutic potential in diseases such as renal ischemia-reperfusion disorders, hypertension, and hypertensive-associated heart disease. Thus, several compounds releasing H_2_S have been described as candidates for the treatment of vascular disease [30] and decreasing platelet-leukocyte aggregation and improving endogenous thrombolysis [31]. Hydrogen sulphide and oestrogen have been shown to inhibit the development of atherosclerosis [32] through upregulating protein S-nitrosylation [33].

Hydrogen sulphide and nitric oxide play a fundamental role as gasotransmitters in the urinary tract, both lower tract and higher tract [34]. In addition, hydrogen sulphide regulates fundamental renal processes such as glomerular filtration and sodium reabsorption, both in the healthy organ and in different pathologies and ischemia [35].

In mammals, the gas is produced from L-cysteine by the three enzymes cystathionine-*ϒ*-lyase (CSE or CGL), cystathionine b-synthase (CBS), and 3-mercaptopyruvate sulphur transferase (MST) [36, 37].

**Figure 2:**
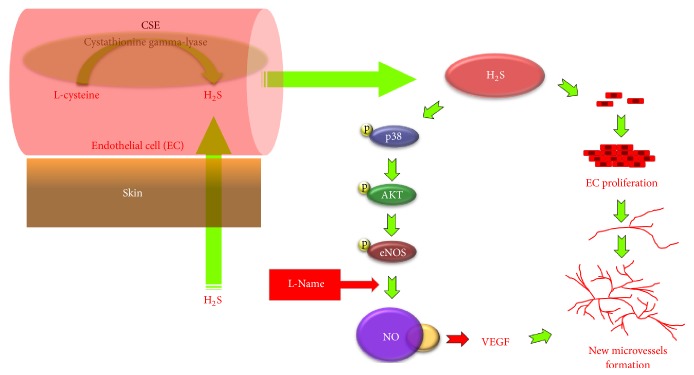
Mechanism of endothelial cell proliferation through hydrogen sulphide activity and NO production by the H_2_S pathway. An increase in vessel formation is produced that is significantly attenuated through NO production blockade by nitro-L-arginine methyl ester (L-NAME).

Hydrogen sulphide-induced relaxation of vascular tissues can be partially reduced by the removal of the vascular endothelium and/or the presence of L-NAME (an inhibitor of NO synthase) [38]. New microvessel formation or angiogenesis occurs as a consequence of these mechanisms and we propose that H_2_S absorbed through the skin can potentiate this effect [39] (Figure 2).

Hydrogen sulphide acts on the migration and survival of endothelial cells to form a network of vessels. This is done through the two genes mitogen-activated protein- (MAP-) kinase-1, an ERK gene (extracellular signal-regulated kinase), and p38, a gene with effects on chaperone heat shock protein 27 (Hsp27) that promotes VEGF secretion. In turn, VEGF inhibits apoptosis, regulating cell development, differentiation, and cell function [40].

There are also three nonenzymatic ways to produce H_2_S: through sulphurous proteins, sulphite, or thiosulphate. Conversely, there are five ways of removing H_2_S in the organism: by its conversion into thiocyanate via rhodanese; by its transformation into sulphurous proteins; through thiol molecules; by converting haemoglobin into sulfhaemoglobin; and by its transformation into methanethiol.

Some human bacteria are able to transform H_2_S into its sulphur derivatives [41]. Thus,* Pseudomonas *spp. transform H_2_S into sulphur (S_0_), S_2_O_6_^2−^, and S_4_O_6_^2−^,* Acinetobacter *spp. into S_0_ and S_2_O_6_^2−^, and* Alcaligenes *spp. and* Ochrobactrum *spp. into S_2_O_6_^2−^ and S_4_O_6_^2−^.

The following factors have been implicated in the storage and release of H_2_S [15]:

1. pH and the conversion of H_2_S into HS^−^ and/or S^2−^.
2. Oxidation/reduction in the presence of oxygen and its conversion into hydrogen thioperoxide and sulphenic, sulphinic, and sulphonic acids, where coenzyme Q-10 plays an important role [42].
3. Hydrogenation and alkalization and transformation in acid labile sulphur (iron-sulphur clusters) and bound sulphane sulphur (persulphite, polysulphite, thiosulphate, etc.).
4. Reduction by microorganisms like sulphate reducing bacteria (SRB) through sulphite reductase a-subunit (CysJ), anaerobic sulphide reductase A (AsrA).
5. Enzymatic synthesis from L-cysteine through the above-described enzymes CSE, CBS, and MST.

Jin et al. [43] described H_2_S as a messenger that acts like a cell signalling molecule on the regulation of blood flow through and with NO and CO; on the protection of organs through phosphoinositide 3-kinase (PI3K), MAP kinase (MAPK), extracellular-signal-regulated kinase (Erk), glycogen synthase kinase 3 beta (GSK3*β*), and protein kinase C (PKC); on inflammation through nuclear factor kappa beta (NF-k*β*); in cellular defences through NF-E2 p45-related factor 2 (Nrf2); as an antiapoptotic factor in growing Bcl-2 cells (B-cell lymphoma 2) and reducing apoptosis by regulating BAX (bcl-2-like protein 4) and caspase-3, influencing cell metabolism in a dose dependent manner and promoting mitochondrial ATP synthesis at low concentrations and inhibition of cytochrome C oxidase at high concentrations; on ion channels producing vasorelaxation via K_ATP_ channels, inflammation through transient receptor potential cation channel subfamily V member 1 (TRPV-1), TRPV-4 [44] and on calcium channels; and through its antioxidation properties acting as a reactive oxygen species (ROS) scavenger and increasing levels of superoxide dismutase (SOD) and glutathione (GSH) [45].

Recently, Bełtowski and Jamroz-Wiśniewska [46] showed that experimentally induced short-term obesity in rats increased endogenous production of hydrogen sulphide in perivascular adipose tissue, while long-term obesity had the opposite effect. These authors also observed that hyperglycaemia suppressed the CSE-H_2_S pathway in adipose tissue and proposed that hydrogen sulphide deficiency could play a role in adipose tissue inflammatory processes associated with obesity or metabolic syndrome.

## 4. Skin Absorption

Sulphurous mineral water may be absorbed through the skin causing vasodilation, analgesia, immune response inhibition, and keratolytic effects that reduce skin desquamation [47]. It is also known that the topical application of H_2_S will also have an effect on the internal organs [48].

The therapeutic action of sulphurous mineral waters is related mainly to sulphur's keratolytic, or peeling, effect. Sulphurous mineral water exerts beneficial anti-inflammatory, keratoplastic, and antipruritic effects [49]. Its bactericidal and antifungal properties have determined its use for the treatment of infected leg ulcers, tinea versicolor, tinea corporis, and tinea capitis [50]. Further, within the epidermis, H_2_S is transformed into sulphur, which may also interact with oxygen radicals in the deeper layers of the epidermis. Here, sulphur may be converted into pentathionic acid (H_2_S_5_O_6_), which could explain the antibacterial and antifungal properties of sulphurous mineral waters [51].

As mentioned above, the pH of sulphurous mineral waters is essential and responsible for its therapeutic effects. Whether effects are superficial or deep depends on the formation of H_2_S on the surface or deep within the skin. Hence, colloidal sulphur acts on the skin surface, HS^−^ is able to penetrate deeper, and H_2_S can either penetrate or be absorbed through the skin reaching the dermis.

## 5. Topical Transformations and Hazards

**Figure 3:**
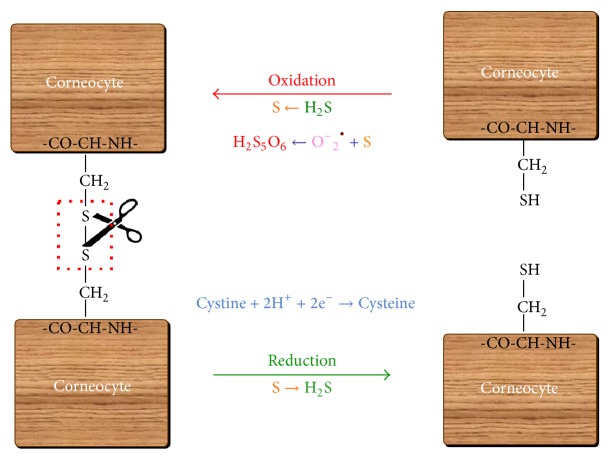
Mechanisms of action of hydrogen sulphide on bonds between corneocytes. Sulphur can be transformed into pentathionic acid and/or produce H_2_S, transforming cystine into cysteine (keratolytic effect). In some situations, hydrogen sulphide can convert cysteine into cystine (keratoplastic effect).

Skin treatments based on mineral water or muds derived from mineral waters have been traditionally used in Europe [52]. Some of the cutaneous activity of sulphurous mineral waters is based on the formation of colloidal sulphur inside the skin via chemical reactions or microbial metabolism, where it then acts as a keratolytic agent. Accordingly, sulphur eliminates disulphide cystine bonds between corneocytes. This gives rise to two cysteine molecules, promoting desquamation of the stratum corneum (Figure 3). The keratolytic or keratoplastic properties of sulphurous mineral waters are support by the following observations:

1. On the skin, H_2_S is converted into sulphur [53].
2. Sulphur may also interact with oxygen radicals in the deeper layers of the epidermis and may in turn be transformed into pentathionic acid or produce H_2_S. This phenomenon was first described in 1913 by McMurtry, who reported that pentathionic acid is a product of oxidized sulphur only when oxygen and water are present. Pentathionic acid is a peptizing agent for sulphur generated by the reaction of H_2_S and sulphur dioxide (SO_2_) [54].
3. In certain conditions, two cysteine molecules may be oxidized to form a new cystine bond [55] and return to their original state [56]. Cysteine residues are also responsible for the quenching of ROS, both within cell protein structures and in the keratinized outer layers of epithelial corneocytes. This quenching leads to the formation of intermolecular and intramolecular S-S bonds in the proteins [57].

In parallel, another study has shown that H_2_S speeds up wound healing in rats with diabetes. The authors related this effect to granulation tissue formation, anti-inflammation, and antioxidant effects, along with increased levels of VEGF [58].

Sulphurous spa baths have been used successfully for immunomediated conditions such as contact dermatitis, psoriasis, and atopic dermatitis, and it has been recently suggested that the active principles of sulphurous mineral waters could play a role in immune regulation in the skin [59].

Sulphurous mineral water inhalations and irrigations have been traditionally used to treat airway diseases [60–63]. According to Keller et al. [64], compared to isotonic saline solution, sulphurous mineral water shows benefits and should be investigated further. It has also demonstrated endogenous production of hydrogen sulphide in human gingival tissue [65] and recent studies suggest that hydrogen sulphide may act as an inhibitory transmitter in esophagus tract [66].

The inhalation of sulphurous mineral waters has been shown to improve the health state of patients with chronic obstructive pulmonary disease (COPD) [67]. The mechanism of these observed effects is thought to be via the antielastase activity of thiol groups which could help control the inflammation associated with upper and lower airway diseases [68].

Topically administered H_2_S plays both physiological and toxicological roles in biological systems. Acute exposure to high levels of H_2_S is life-threatening, while long-term exposure to environmental levels has detrimental effects on human health. As an example, concentrations higher than 25 ppm in the environment have been linked to eye damage and chronic exposure to hydrogen sulphide has been attributed to severe effects on health [69]. The acute inhalation of H_2_S at levels above 50–100 ppm is considered harmful and breathing problems arise when exposure is chronic at 10–20 ppm [70].

Emergency hospital visits for heart disease often accompanied by respiratory disease or stroke have been linked to same-day hydrogen sulphide concentrations exceeding 7.00 *μ*g/m^3^. This correlation was more pronounced among males and those 73 years and older than among females and younger individuals [71].

Long-term exposure to even low levels of H_2_S at the workplace has been reported to raise mean methaemoglobin and sulfhaemoglobin levels [72].

The topical use of H_2_S is not free of complications. The use of concentrated sulphurous mineral water and especially bathing in hot springs can produce skin conditions ranging from irritative dermatitis to chemical burns.

Lee and Wu [73] described the case of a 65-year-old man with no history of dermatologic disease, who suddenly developed painful, confluent round ulcers on his legs after bathing in a hot sulphurous spring, which had been a regular habit for the past 10 years. In a skin biopsy, these lesions were attributed to epidermal necrosis accompanied by numerous neutrophils typical of a chemical burn.

In contrast, Ferreira et al. [74] described that some sulphurous mineral waters are anti-irritants and may help relieve skin irritation. In dermatologic formulations, they may also improve the tolerability of the products.

## 6. Cell and Immunological Messengers

**Figure 4:**
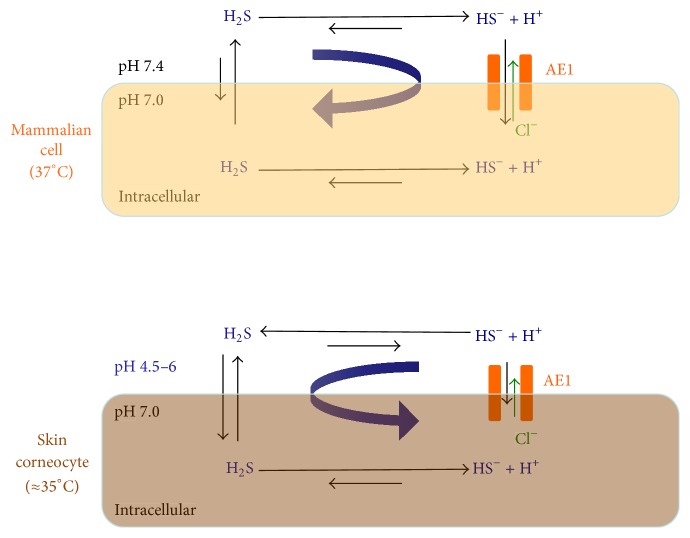
Cellular uptake of hydrogen sulphide according to pH and temperature. In cells, sulphur is mostly absorbed as bisulphide salt, while, on skin, hydrogen sulphide is the main molecule involved in the absorption and release of sulphur.

In mammalian cells, H_2_S passes through the cell membrane and its salts pass through bisulphide channels, where they are exchanged for Cl^−^ (by anion exchange protein AE1). In the extracellular matrix, under physiological conditions of pH 7.4 and 37°C, only approximately 20% H_2_S exists as a gas. H_2_S dissociates to HS^−^ with a trace amount of S^2−^. This means it is mostly absorbed as bisulphide (Figure 4).

Hydrogen sulphide is released from the cell and then dissociates in the extracellular environment where the pH is slightly higher than inside the cell. The reaction balance is mainly in this direction.

In bacteria, the extracellular environment pH is slightly lower than inside the cell. While H_2_S enters cells through the plasma membrane, HS^−^ is released through bisulphide channels to the extracellular environment. This time, the reaction occurs in the opposite direction [75].

However, within the epidermis, the extracellular environmental pH is much lower than inside the cell (4.5–6.5) and the temperature is also slightly lower (35°C). Thus, H_2_S is the main molecule involved in the absorption and release of sulphur.

**Figure 5:**
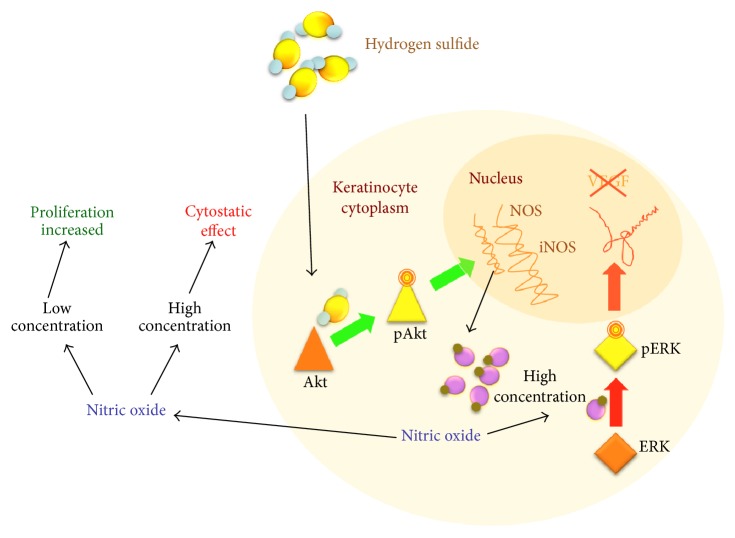
Hydrogen sulphide has a dual effect on keratinocytes: at low concentrations, it induces their proliferation, while, at high concentrations, it exerts a cytostatic effect on these cells and reduces VEGF release via the ERK pathway.

Merighi et al. [76] conducted a study in which it was shown for the first time that H_2_S significantly increases NO levels in a protein kinase B (Akt) gene-dependent manner. Inside keratinocytes, NO is synthesized from arginine at low concentrations of H_2_S by constitutive nitric oxide synthases (NOS) and at higher concentrations by inducible nitric oxide synthases (iNOS). Hence any increase in NO concentration reduces ERK1/2 activation with the consequence of diminished VEGF release [77] (Figure 5). According to these authors, the proliferation of keratinocytes is enhanced by their exposure to low H_2_S concentrations, while high concentrations of H_2_S lead to considerably increased NO concentrations, exerting a cytostatic effect on these cells. Other authors have observed that, in psoriasis, despite apparently elevated iNOS, the levels of NO produced were inadequate to induce cell differentiation, although sufficient to promote proliferation. Increasing the concentrations of NO in the epidermis will promote keratinocyte differentiation rather than proliferation [43].

These observations suggest promising therapeutic applications of H_2_S releasing agents for chronic inflammatory disorders of the skin such as psoriasis owing to its NO donor and anti-VEGF properties. However, such compounds still need to be assessed in a clinical setting.

According to Jin et al. [43], H_2_S shows dual behaviour depending on its concentration outside the cell. At low concentrations, it stimulates keratinocyte proliferation (antiapoptotic) and has provasodilation and proangiogenesis properties, while, at high concentrations, it promotes keratinocyte differentiation (proapoptotic) and shows antivasodilation and antiangiogenesis properties.

Yang et al. [78] reported that the newly synthesized H_2_S donor is capable of protecting human skin keratinocytes from methylglyoxal-induced injury and dysfunction. Thus, it could be that H_2_S-releasing molecules will improve wound healing in patients with diabetes mellitus. Suzuki et al. [79] have found differences in plasma levels of hydrogen sulphide in diabetic patients.

Under appropriate conditions, H_2_S reduces clonal growth, cell proliferation, and the adhesion of mature human keratinocytes by limiting the keratinocyte stem cell subpopulation in culture [80].

Some mineral waters have effects on the immune system. Different mineral waters seem to exert some immunomodulation activity and to increase levels of beta-endorphins in human skin. Unfortunately, these “miraculous” effects are still poorly documented and the lack of double-blinded studies precludes any generalizations about mineral waters as cutaneous prescriptions [81].

Hydrogen sulphide inhibits IL-8 expression in human keratinocytes via MAP kinase signalling. Along with its known anti-inflammatory activity, this could help explain some of the biological effects of sulphurous mineral water therapy [82] including Langerhans cell migration [83].

Mirandola et al. [84] reported that mitogen-activated protein is able to diminish the IL-2 production and cell cytotoxic response shown by peripheral blood lymphocytes and therefore mitigates local inflammatory responses and cell toxicity as characteristic anti-inflammatory effects of sulphur compounds.

Rinaldi et al. [85] assessed the in vitro effects of H_2_S on the survival capacity and bactericidal actions of human neutrophils. These authors proposed the following:

1. Bisulphide ions favour the survival of granulocytes cultured in serum-free medium but not the survival of lymphocytes or eosinophils.
2. This prosurvival effect is mediated through caspase-3 cleavage inhibition and p38 MAP-kinase phosphorylation.
3. The bactericidal actions of neutrophils are not modified by H_2_S. They concluded that bisulphide promotes the short-term survival of neutrophils and that this could speed up the resolution of inflammation and prevent further inflammation.

Inflammation is a response to traumatic, infectious, postischemic, toxic, or autoimmune damage. However, uncontrolled inflammation can lead to disease, and inflammation is now believed to be responsible for several disease conditions.

Bhatia [86] reported that H_2_S acts as a novel mediator of inflammation. Soria et al. [87] determined that sulphurous mineral water intake improves haematological and biochemical markers of muscle damage produced in response to endurance exercise in well-trained athletes and thus may protect from exercise-induced muscle damage.

Sulphurous mineral waters may help manage chronic inflammatory and age-related disorders through their combined anti-inflammatory and antioxidant properties [88].

Transient receptor potential ankyrin 1 (TRPA1), activated by H_2_S, plays an important role in chronic arthritis/osteoarthritis and related pain behaviours in the mouse. This receptor may therefore be a promising target for novel analgesic/anti-inflammatory drugs [89]. The interaction of hydrogen sulphide on T-Type Ca^2+^ channels can serve as a therapeutic target for the treatment of intractable pain and neuronal lesions [90].

Hydrogen sulphide decreases IL-1*β*-induced activation of fibroblast-like synoviocytes obtained from patients with osteoarthritis [91] and is thought to delay cartilage destruction in part by reducing activation of the nuclear factor NFkB [92].

Finally, Benedetti et al. [93] suggested that, to ensure long-lasting chondroprotective effects of sulphur-based therapies, standard mud bath treatments should be associated with hydropinotherapy to keep reduced oxidative, inflammatory, and degradative stimuli for longer.

## 7. Discussion

H_2_S induces a wide range of physiological responses such as blood pressure modulation, protection against ischemic reperfusion injury, and anti-inflammatory reactions.

It is well documented that H_2_S is the main factor responsible for capillary vasodilation. Accordingly, the exogenous administration of H_2_S elicits vascular relaxation in animal preparations both in vitro and in vivo [94]. Exogenous H_2_S could protect arterial endothelial cells [95]. Mancini et al. [96] described additional benefits of balneokinetic treatment with sulphurous mineral water over elastic compression in patients with symptomatic varices. According to these authors, the improvement in the venoarteriolar reflex observed could support these subjective benefits.

Sulphurous mineral water-based balneotherapy significantly reduces limb spasticity and pain and can help in the treatment of poststroke patients [97]. These observations are compatible with the regeneration and anti-inflammatory activity of sulphurous mineral waters [98] and may be essential for Schwann cell responses to peripheral nerve injury [99].

Observations so far strongly suggest that H_2_S modulation could have therapeutic benefits. Hence, sulphurous mineral waters are not only useful research tools but also show promise as therapeutic agents [100].

Intriguingly too, H_2_S has been reported to regulate cell cycle and survival in healthy cells which suggests that it may regulate cell fate and hence the ageing process [101].

Intense research effort is also being devoted to addressing the use of H_2_S-donor drugs capable of the controlled release of H_2_S into the bloodstream. Notwithstanding, according to Haouzi [102], the levels of H2S tested in vitro are toxic and the low levels that will need to be administered in vivo to avoid any toxic effects are unlikely to give rise to adequate H_2_S concentrations in tissues.

Pharmacological molecules that release H_2_S too quickly do not adequately mimic the physiological effects of the gas. Hence, slow-releasing H_2_S donors are a more appropriate source of hydrogen sulphide than even a sulphur salt solution. In addition, the beneficial effects of H_2_S on inflammation are concentration and time-dependent and may involve bell-shaped dose-response curves.

The therapeutic properties of mineral-medicinal waters are determined by their inherent properties. This means they cannot be chemically modified but can be modified discretely by simple physical means. Sulphurous mineral water springs used in balneotherapy tend to have low sulphide concentrations. In Spain, such sulphide concentrations are 45 mg/l maximum and are therefore not considered toxic.

Besides an attenuating effect of slow skin absorption, the topical application of sulphurous mineral waters in well-ventilated and controlled environments such as spas can be considered safe. In these environments, treatment duration and chronicity can be modulated in addition to the active concentrations of sulphurous mineral water. In this manner, sulphide release by sulphurous mineral waters can be to a certain extent controlled.

In a review of studies assessing the clinical use of H_2_S, Bełtowski [103] describes clinical applications of the gas in the following conditions: pulmonary hypertension, arterial hypertension, atherosclerosis, ischemia-reperfusion injury, heart failure, peptic ulcer, acute and chronic inflammatory diseases, Parkinson's disease, Alzheimer's disease, erectile dysfunction, and skin diseases.

In conclusion, several new lines of evidence indicating a role of hydrogen sulphide as a cell messenger with cytoprotective effects anticipate promising perspectives for treatments with sulphurous mineral waters [104].

## References

[1] Stier-Jarmer M., Kus S., Frisch D., Sabariego C., Schuh A. (2015). Health resort medicine in non-musculoskeletal disorders: is there evidence of its effectiveness?. *International Journal of Biometeorology*.

[2] Sen N. (2017). Functional and molecular insights of hydrogen sulfide signaling and protein sulfhydration. *Journal of Molecular Biology*.

[3] Gutenbrunner C., Bender T., Cantista P., Karagülle Z. (2010). A proposal for a worldwide definition of health resort medicine, balneology, medical hydrology and climatology. *International Journal of Biometeorology*.

[4] Vaccarezza M., Vitale M. (2010). Crenotherapy: a neglected resource for human health now re-emerging on sound scientific concepts. *International Journal of Biometeorology*.

[5] Fioravanti A., Cantarini L., Guidelli G. M., Galeazzi M. (2011). Mechanisms of action of spa therapies in rheumatic diseases: what scientific evidence is there?. *Rheumatology International*.

[6] Tenti S., Cheleschi S., Galeazzi M., Fioravanti A. (2015). Spa therapy: can be a valid option for treating knee osteoarthritis?. *International Journal of Biometeorology*.

[7] Varga C. (2016). On the proper study design applicable to experimental balneology. *International Journal of Biometeorology*.

[8] Carretero M. I. (2002). Clay minerals and their beneficial effects upon human health. A review. *Applied Clay Science*.

[9] Gomes C., Carretero M. I., Pozo M. (2013). Peloids and pelotherapy: historical evolution, classification and glossary. *Applied Clay Science*.

[10] Pozo M., Carretero M. I., Maraver F. (2013). Composition and physico-chemical properties of peloids used in Spanish spas: a comparative study. *Applied Clay Science*.

[11] Maraver F., Fernández-Torán M. Á., Corvillo I. (2015). Pelotherapy, a review. *Medicina Naturista*.

[12] Goddard A. L., Lio P. A. (2015). Alternative, complementary, and forgotten remedies for atopic dermatitis. *Evidence-based Complementary and Alternative Medicine*.

[13] Carbajo J. M. (2014). *Evaluación de los cambios en la piel tras la aplicación de cosméticos elaborados a partir del sedimento de las aguas minero-medicinales Lanjarón-Capuchina mediante métodos de bioingenieria cutánea [Ph.D. thesis]*.

[14] Carbajo J. M., Maraver F. Hydrogen sulfide and health. New insights.

[15] Kolluru G. K., Shen X., Bir S. C., Kevil C. G. (2013). Hydrogen sulfide chemical biology: pathophysiological roles and detection. *Nitric Oxide*.

[16] Chen K. Y., Morris J. C. (1972). Kinetics of oxidation of aqueous sulfide by oxygen. *Environmental Science and Technology*.

[17] Mitchell S. C., Waring R. H. (2016). Sulphate absorption across biological membranes. *Xenobiotica*.

[18] Barton L. L., Fardeau M. L., Fauque G. D. (2014). Hydrogen sulfide: a toxic gas produced by dissimilatory sulfate and sulfur reduction and consumed by microbial oxidation. *The Metal-Driven Biogeochemistry of Gaseous Compounds in the Environment*.

[19] Bağdatlı A. O., Donmez A., Eröksüz R., Bahadır G., Turan M., Erdoğan N. (2015). Does addition of ‘mud-pack and hot pool treatment’ to patient education make a difference in fibromyalgia patients? A randomized controlled single blind study. *International Journal of Biometeorology*.

[20] Armijo F., Maraver F., Carretero M. I. (2015). The water effect on instrumental hardness and adhesiveness of clay mixtures for pelotherapy. *Applied Clay Science*.

[21] Armijo F., Maraver F., Pozo M. (2016). Thermal behaviour of clays and clay-water mixtures for pelotherapy. *Applied Clay Science*.

[22] Fortunati N. A., Fioravanti A., Seri G., Cinelli S., Tenti S. (2016). May spa therapy be a valid opportunity to treat hand osteoarthritis? A review of clinical trials and mechanisms of action. *International Journal of Biometeorology*.

[23] Bender T., Karagülle Z., Bálint G. P., Gutenbrunner C., Bálint P. V., Sukenik S. (2005). Hydrotherapy, balneotherapy, and spa treatment in pain management. *Rheumatology International*.

[24] Forestier R., Desfour H., Tessier J.-M. (2010). Spa therapy in the treatment of knee osteoarthritis: a large randomised multicentre trial. *Annals of the Rheumatic Diseases*.

[25] Costantino M., Filippelli A., Quenau P., Nicolas J.-P., Coiro V. (2012). Sulphur mineral water and SPA therapy in osteoarthritis. *Therapie*.

[26] Santos I., Cantista P., Vasconcelos C. (2016). Balneotherapy in rheumatoid arthritis—a systematic review. *International Journal of Biometeorology*.

[27] Kimura H. (2014). Hydrogen sulfide and polysulfides as biological mediators. *Molecules*.

[28] Koike S., Ogasawara Y. (2016). Sulfur atom in its bound state is a unique element involved in physiological functions in mammals. *Molecules*.

[29] Abe K., Kimura H. (1996). The possible role of hydrogen sulfide as an endogenous neuromodulator. *Journal of Neuroscience*.

[30] Holwerda K. M., Karumanchi S. A., Lely A. T. (2015). Hydrogen sulfide: role in vascular physiology and pathology. *Current Opinion in Nephrology & Hypertension*.

[31] Grambow E., Leppin C., Leppin K. (2016). The effects of hydrogen sulfide on platelet–leukocyte aggregation and microvascular thrombolysis. *Platelets*.

[32] Li H., Mani S., Wu L. (2017). The interaction of estrogen and CSE/H2S pathway in the development of atherosclerosis. *American Journal of Physiology—Heart and Circulatory Physiology*.

[33] Lin Y., Chen Y., Zhu N., Zhao S., Fan J., Liu E. (2016). Hydrogen sulfide inhibits development of atherosclerosis through up-regulating protein S-nitrosylation. *Biomedicine and Pharmacotherapy*.

[34] Fernandes V. S., Hernández M. (2016). The role of nitric oxide and hydrogen sulfide in urinary tract function. *Basic & Clinical Pharmacology & Toxicology*.

[35] Feliers D., Lee H. J., Kasinath B. S. (2016). Hydrogen sulfide in renal physiology and disease. *Antioxidants & Redox Signaling*.

[36] Zhao Y., Biggs T. D., Xian M. (2014). Hydrogen sulfide (H2S) releasing agents: chemistry and biological applications. *Chemical communications*.

[37] Rose P., Moore P. K., Zhu Y. Z. (2017). H_2_S biosynthesis and catabolism: new insights from molecular studies. *Cellular and Molecular Life Sciences*.

[38] Altaany Z., Yang G., Wang R. (2013). Crosstalk between hydrogen sulfide and nitric oxide in endothelial cells. *Journal of Cellular and Molecular Medicine*.

[39] Katsouda A., Bibli S.-I., Pyriochou A., Szabo C., Papapetropoulos A. (2016). Regulation and role of endogenously produced hydrogen sulfide in angiogenesis. *Pharmacological Research*.

[40] Sanmartín C., Plano D., Font M., Palop J. A. (2011). Kinase regulation by sulfur and selenium containing compounds. *Current Cancer Drug Targets*.

[41] Pacheco-Aguilar J. R., Maldonado-Vega M., Peña-Cabriales J. J. (2012). Metabolismo del azufre de aislados bacterianos provenientes de un humedal artificial empleado para el tratamiento de efluentes de la industria curtidora. *Revista Internacional de Contaminación Ambiental*.

[42] Ziosi M., Di Meo I., Kleiner G. (2017). Coenzyme Q deficiency causes impairment of the sulfide oxidation pathway. *EMBO Molecular Medicine*.

[43] Jin Z., Chan H., Ning J., Lu K., Ma D. (2015). The role of hydrogen sulfide in pathologies of the vital organs and its clinical application. *Journal of Physiology and Pharmacology*.

[44] Naik J. S., Osmond J. M., Walker B. R., Kanagy N. L. (2016). Hydrogen sulfide-induced vasodilation mediated by endothelial TRPV4 channels. *American Journal of Physiology—Heart and Circulatory Physiology*.

[45] Olas B. (2017). Hydrogen sulfide as a ‘double-faced’ compound: one with pro- and antioxidant effect. *Advances in Clinical Chemistry*.

[46] Bełtowski J., Jamroz-Wiśniewska A. (2017). Hydrogen sulfide in the adipose tissue—physiology, pathology and a target for pharmacotherapy. *Molecules*.

[47] Nasermoaddeli A., Kagamimori S. (2005). Balneotherapy in medicine: a review. *Environmental Health and Preventive Medicine*.

[48] Sukenik S., Buskila D., Neumann L., Kleiner-Baumgarten A., Zimlichman S., Horowitz J. (1990). Sulphur bath and mud pack treatment for rheumatoid arthritis at the Dead Sea area. *Annals of the Rheumatic Diseases*.

[49] Rodrigues L., Ekundi-Valentim E., Florenzano J. (2017). Protective effects of exogenous and endogenous hydrogen sulfide in mast cell-mediated pruritus and cutaneous acute inflammation in mice. *Pharmacological Research*.

[50] Parish L. C., Witkowski J. A. (1994). Dermatologic balneology: the American view of waters, spas, and hot springs. *Journal of the European Academy of Dermatology and Venereology*.

[51] Matz H., Orion E., Wolf R. (2003). Balneotherapy in dermatology. *Dermatologic Therapy*.

[52] Lotti T., Freedman D. (1994). Balneology and spa treatments in dermatology: the European point of view. *Journal of the European Academy of Dermatology and Venereology*.

[53] Lin A. N., Reimer R. J., Carter D. M. (1988). Sulfur revisited. *Journal of the American Academy of Dermatology*.

[54] Young H. C. (1928). Pentathionic acid, the fungicidal factor of sulphur. *Science*.

[55] Wani R., Nagata A., Murray B. W. (2014). Protein redox chemistry: post-translational cysteine modifications that regulate signal transduction and drug pharmacology. *Frontiers in Pharmacology*.

[56] Jones D. P., Go Y.-M., Anderson C. L., Ziegler T. R., Kinkade J. M., Kirlin W. G. (2004). Cysteine/cystine couple is a newly recognized node in the circuitry for biologic redox signaling and control. *The FASEB Journal*.

[57] Vermeij W. P., Alia A., Backendorf C. (2011). ROS quenching potential of the epidermal cornified cell envelope. *The Journal of Investigative Dermatology*.

[58] Wang G., Li W., Chen Q., Jiang Y., Lu X., Zhao X. (2015). Hydrogen sulfide accelerates wound healing in diabetic rats. *International Journal of Clinical and Experimental Pathology*.

[59] Gálvez Galve J. J., Saz Peiró P., Ortiz Lucas M., Torres A. H., Gil E. S., Pérez M. B. (2012). Quality of life and assessment after local application of sulphurous water in the home environment in patients with psoriasis vulgaris: a randomised placebo-controlled pilot study. *European Journal of Integrative Medicine*.

[60] Costantino M. (2008). The rhinogenic deafness and SPA therapy: clinical-experimental study. *La Clinica Terapeutica*.

[61] Salami A., Dellepiane M., Crippa B. (2008). Sulphurous water inhalations in the prophylaxis of recurrent upper respiratory tract infections. *International Journal of Pediatric Otorhinolaryngology*.

[62] Salami A., Dellepiane M., Strinati F., Guastini L., Mora R. (2010). Sulphurous thermal water inhalations in the treatment of chronic rhinosinusitis. *Rhinology*.

[63] Varricchio A., Giuliano M., Capasso M. (2013). Salso-sulphide thermal water in the prevention of recurrent respiratory infections in children. *International Journal of Immunopathology and Pharmacology*.

[64] Keller S., König V., Mösges R. (2014). Thermal water applications in the treatment of upper respiratory tract diseases: a systematic review and meta-analysis. *Journal of Allergy*.

[65] Chun-Mei J., Wu C., Guo-Liang M., Yue G., Ning C., Ji Y. (2017). Production of endogenous hydrogen sulfide in human gingival tissue. *Archives of Oral Biology*.

[66] Zhang L., Zhao W., Zheng Z. (2016). Hydrogen sulfide synthesis enzymes reduced in lower esophageal sphincter of patients with achalasia. *Diseases of the Esophagus*.

[67] Contoli M., Gnesini G., Forini G. (2013). Reducing agents decrease the oxidative burst and improve clinical outcomes in COPD patients: a randomised controlled trial on the effects of sulphurous thermal water inhalation. *The Scientific World Journal*.

[68] Braga P. C., Dal Sasso M., Culici M. (2010). Effects of sulphurous water on human neutrophil elastase release. *Therapeutic Advances in Respiratory Disease*.

[69] Lambert T. W., Goodwin V. M., Stefani D., Strosher L. (2006). Hydrogen sulfide (H_2_S) and sour gas effects on the eye. A historical perspective. *Science of the Total Environment*.

[70] Lewis R. J., Copley G. B. (2015). Chronic low-level hydrogen sulfide exposure and potential effects on human health: a review of the epidemiological evidence. *Critical Reviews in Toxicology*.

[71] Finnbjornsdottir R. G., Carlsen H. K., Thorsteinsson T. (2016). Association between daily hydrogen sulfide exposure and incidence of emergency hospital visits: a population-based study. *PLoS ONE*.

[72] Saeedi A., Najibi A., Mohammadi-Bardbori A. (2015). Effects of long-term exposure to hydrogen sulfide on human red blood cells. *International Journal of Occupational and Environmental Medicine*.

[73] Lee C.-C., Wu Y.-H. (2014). Sulfur spring dermatitis. *Cutis*.

[74] Ferreira M. O., Costa P. C., Bahia M. F. (2010). Effect of São Pedro do sul thermal water on skin irritation. *International Journal of Cosmetic Science*.

[75] Kimura H. (2015). Hydrogen sulfide and polysulfides as signaling molecules. *Proceedings of the Japan Academy Series B: Physical and Biological Sciences*.

[76] Merighi S., Gessi S., Varani K., Fazzi D., Borea P. A. (2012). Hydrogen sulfide modulates the release of nitric oxide and VEGF in human keratinocytes. *Pharmacological Research*.

[77] Szabo C. (2017). Hydrogen sulfide, an enhancer of vascular nitric oxide signaling: mechanisms and implications. *American Journal of Physiology—Cell Physiology*.

[78] Yang C.-T., Zhao Y., Xian M. (2014). A novel controllable hydrogen sulfide-releasing molecule protects human skin keratinocytes against methylglyoxal-induced injury and dysfunction. *Cellular Physiology and Biochemistry*.

[79] Suzuki K., Sagara M., Aoki C., Tanaka S., Aso Y. (2017). Clinical implication of plasma hydrogen sulfide levels in Japanese patients with type 2 diabetes. *Internal Medicine*.

[80] Gobbi G., Ricci F., Malinverno C. (2009). Hydrogen sulfide impairs keratinocyte cell growth and adhesion inhibiting mitogen-activated protein kinase signaling. *Laboratory Investigation*.

[81] Ghersetich I., Lotti T. M. (1996). Immunologic aspects: immunology of mineral water spas. *Clinics in Dermatology*.

[82] Mirandola P., Gobbi G., Micheloni C. (2011). Hydrogen sulfide inhibits IL-8 expression in human keratinocytes via MAP kinase signaling. *Laboratory Investigation*.

[83] Mustak M., Neumüller J. (2005). Influence of sulphur water on skin explants and associated effects on the migration and the production of TNF-*α* by Langerhans cells. *Physikalische Medizin Rehabilitationsmedizin Kurortmedizin*.

[84] Mirandola P., Gobbi G., Sponzilli I. (2007). Exogenous hydrogen sulfide induces functional inhibition and cell death of cytotoxic lymphocytes subsets. *Journal of Cellular Physiology*.

[85] Rinaldi L., Gobbi G., Pambianco M., Micheloni C., Mirandola P., Vitale M. (2006). Hydrogen sulfide prevents apoptosis of human PMN via inhibition of p38 and caspase 3. *Laboratory Investigation*.

[86] Bhatia M. (2015). H_2_S and inflammation: an overview. *Chemistry, Biochemistry and Pharmacology of Hydrogen Sulfide*.

[87] Soria M., González-Haro C., Esteva S., Escanero J. F., Pina J. R. (2014). Effect of sulphurous mineral water in haematological and biochemical markers of muscle damage after an endurance exercise in well-trained athletes. *Journal of Sports Sciences*.

[88] Prandelli C., Parola C., Buizza L. (2013). Sulphurous thermal water increases the release of the anti-inflammatory cytokine IL-10 and modulates antioxidant enzyme activity. *International Journal of Immunopathology and Pharmacology*.

[89] Horváth Á., Tékus V., Boros M. (2016). Transient receptor potential ankyrin 1 (TRPA1) receptor is involved in chronic arthritis: in vivo study using TRPA1-deficient mice. *Arthritis Research and Therapy*.

[90] Fukami K., Sekiguchi F., Kawabata A. (2016). Hydrogen sulfide and T-type Ca^2+^ channels in pain processing, neuronal differentiation and neuroendocrine secretion. *Pharmacology*.

[91] Sieghart D., Liszt M., Wanivenhaus A. (2015). Hydrogen sulphide decreases IL-1*β*-induced activation of fibroblast-like synoviocytes from patients with osteoarthritis. *Journal of Cellular and Molecular Medicine*.

[92] Burguera E. F., Vela-Anero Á., Magalhães J., Meijide-Faílde R., Blanco F. J. (2014). Effect of hydrogen sulfide sources on inflammation and catabolic markers on interleukin 1*β*-stimulated human articular chondrocytes. *Osteoarthritis and Cartilage*.

[93] Benedetti S., Canino C., Tonti G. (2010). Biomarkers of oxidation, inflammation and cartilage degradation in osteoarthritis patients undergoing sulfur-based spa therapies. *Clinical Biochemistry*.

[94] Kutz J. L., Greaney J. L., Santhanam L., Alexander L. M. (2015). Evidence for a functional vasodilatatory role for hydrogen sulphide in the human cutaneous microvasculature. *Journal of Physiology*.

[95] Liu J., Wu J., Sun A. (2016). Hydrogen sulfide decreases high glucose/palmitate-induced autophagy in endothelial cells by the Nrf2-ROS-AMPK signaling pathway. *Cell and Bioscience*.

[96] Mancini S., Piccinetti A., Nappi G., Mancini S., Caniato A., Coccheri S. (2003). Clinical, functional and quality of life changes after balneokinesis with sulphurous water in patients with varicose veins. *Vasa*.

[97] Erceg-Rukavina T., Stefanovski M. (2015). Balneotherapy in treatment of spastic upper limb after stroke. *Medical Archives*.

[98] Faga A., Nicoletti G., Gregotti C., Finotti V., Nitto A., Gioglio L. (2012). Effects of thermal water on skin regeneration. *International Journal of Molecular Medicine*.

[99] Park B. S., Kim H.-W., Rhyu I. J. (2015). Hydrogen sulfide is essential for Schwann cell responses to peripheral nerve injury. *Journal of Neurochemistry*.

[100] Zhao Y., Pacheco A., Xian M. (2015). Medicinal chemistry: insights into the development of novel H_2_S donors. *Chemistry, Biochemistry and Pharmacology of Hydrogen Sulfide*.

[101] Qabazard B., Stürzenbaum S. R. (2015). H_2_S: a new approach to lifespan enhancement and healthy ageing?. *Chemistry, Biochemistry and Pharmacology of Hydrogen Sulfide*.

[102] Haouzi P. (2016). Is exogenous hydrogen sulfide a relevant tool to address physiological questions on hydrogen sulfide?. *Respiratory Physiology and Neurobiology*.

[103] Bełtowski J. (2015). Hydrogen sulfide in pharmacology and medicine—an update. *Pharmacological Reports*.

[104] Varga C. (2012). Balneoprevention: new approaches. *International Journal of Biometeorology*.

